# A Bifunctional Fluorescence Probe Based on AIE-ICT Strategy for Visual Detection of Cu^2+^/Co^2+^ in Complex Matrix

**DOI:** 10.3390/molecules28052059

**Published:** 2023-02-22

**Authors:** Jingtao Zhao, Jiaxin Lian, Wenyue Pan, Jialing Du, Ziteng Liu, Longshan Zhao

**Affiliations:** School of Pharmacy, Shenyang Pharmaceutical University, Shenyang 110016, China

**Keywords:** aggregation-induced emission, fluorescent probe, copper (II) and cobalt (II), glutathione, Schiff base

## Abstract

A novel fluorescence chemical sensor-based probe 1-{[(E)-(2-aminophenyl)azanylidene]methyl}naphthalen-2-ol (AMN) was designed and synthesized, which performed a “naked eye” detection ability toward Cu^2+^ and Co^2+^ based on aggregation-induced emission (AIE) fluorescence strategy. It has sensitive detection ability for Cu^2+^ and Co^2+^. In addition, the color changed from yellow-green to orange under the sunlight, realizing the rapid identification of Cu^2+^/Co^2+^, which has the potential of on-site visual detection under the “naked eye”. Moreover, different “on” and “off” fluorescence expressions were exhibited under excessive glutathione (GSH) in AMN-Cu^2+^ and AMN-Co^2+^ systems, which could be employed to distinguish Cu^2+^ from Co^2+^. The detection limits for Cu^2+^ and Co^2+^ were measured to be 8.29 × 10^−8^ M and 9.13 × 10^−8^ M, respectively. The binding mode of AMN was calculated to be 2:1 by Jobs’ plot method analysis. Ultimately, the new fluorescence sensor was applied to detect Cu^2+^ and Co^2+^ in real samples (tap water, river water, and yellow croaker), and the results were satisfying. Therefore, this high-efficiency bifunctional chemical sensor platform based on “on–off” fluorescence detection will provide significant guidance for the advance development of single-molecule sensors for multi-ion detection.

## 1. Introduction

Metals are considered to potential environmental contaminants owing to their toxicity towards ecology and human health. Among kinds of heavy metal ions, copper (Cu^2+^) and cobalt (Co^2+^) ions receive considerable attention because of their facile accumulation in living systems via the food chain and disturbing the biological pathways of various living systems [1]. Cu^2+^ plays significant biological roles in human body [2,3] and is associated with numerous metabolic catalytic reactions [4,5,6]. Deficiencies of Cu^2+^ result in serious health disorders, including fatigue and anemia, etc. [7]. However, excesses of Cu^2+^ in the body also lead to increasing the risk of neurological diseases such as Alzheimer’s [8], Parkinson’s, and Wilson’s disease [9,10,11,12]. In contrast, Co^2+^ is the cofactor of vitamin B12 (cyanocobalamin), which performs vital functions in the metabolism of folic acid and fatty acids [13]. According to the regulations of the US Environmental Protection Agency (EPA), the allowable limit of cobalt in air is 0.0004 µg/m^3^ and 2 µg/L in drinking water [14]. However, excessive exposure to cobalt may lead to hypothyroidism, pulmonary fibrosis, and asthma [15,16]. Consequently, given the importance of heavy metal ions, numerous efforts have been developed on efficient detection methods for Cu^2+^ and Co^2+^ [17,18,19].

Compared with traditional chemosensors, including atomic absorption spectrometry (AAS) [20], inductively coupled plasma mass spectrometry (ICP-MS), colorimetric analysis [21], electrochemical analysis [22], and enzyme-linked immune sorbent assay (ELISA) [23,24], fluorescence chemosensors have attracted more attention owing to the remarkable strengths of high sensitivity and selectivity, as well as undamaged and swift real-time detection [25,26,27,28]. Contemporarily, many fluorescent probes have been widely reported for the independent detection of Cu^2+^ [29,30] or Co^2+^ [31], but few have been reported for the simultaneous detection of Cu^2+^ and Co^2+^. In fact, heavy metals and metals usually coexist in low concentrations in environmental and biological systems. Therefore, it is important to develop multifunctional fluorescent probes that could detect both Cu^2+^ and Co^2+^.

A key point is to combine different functional groups subtly in the design of a probe. A lot of traditional dyes have been devised as prominent fluorescence probes with a large planar π-π-conjugated structure, such as BODIPY, fluorescein, rhodamine, and cyanine [32,33,34,35,36]. However, the above-mentioned organic fluorescent groups have bright fluorescence levels when in diluted solution, while in high-concentration solution, the fluorescence gets weaker and even disappears, which is called an aggregation-caused quenching (ACQ) effect. This extremely restricts the application in reality. Tang’s team revealed the aggregation-induced emission (AIE) phenomenon for the first time, which efficaciously avoided the ACQ effect [37,38]. Contrary to the ACQ phenomenon, AIE exhibited extremely bright fluorescence under the aggregated state, which resulted from the restriction of intramolecular motion [39]. Due to its unique properties, including high fluorescence efficiency and photostability, diverse molecular structure design, and flexible detection mode, AIE has been successfully applied in the fields of detection, disease diagnosis, and therapy [40,41,42]. Therefore, AIE materials have become one of the most popular fluorescence probe materials and are receiving more and more attention. Schiff bases, based on AIE characteristics, eliminate the emergence of the ACQ effect. The fluorescent probes based on Schiff bases, which feature simple synthesis, light stability, and low cost of raw materials, were obtained from the condensation reaction between aldehydes and amines. The determination could be carried out smoothly attributing to the easy combination of hydroxyl aromatic aldehyde Schiff base compounds containing rich electronic nitrogen and electron-deficient metal ions [43,44]. When the compound chooses a solvent that is easy to dissolve, the C-N bond of it can rotate freely [45]. Thus, attenuation of nonradiative energy results in weak fluorescence emission. Conversely, when the compound is presented in a solvent with bad solubility, it could generate an AIE effect, leading to an increase in fluorescence intensity, which is mediated by intramolecular hydrogen bonds between hydroxyl and nitrogen atoms. This indicates the activation of excited-state intramolecular proton transfer.

Herein, 1-{[(E)-(2-aminophenyl) azanylidene] methyl} naphthalen-2-ol (AMN) was designed and synthesized based on the condensation reaction of 2-hydroxy-1-naphthalene formaldehyde and o-phenylenediamine. In DMSO-PBS (*v*/*v* = 3:2, pH 6.8) mixed solution at room temperature, AMN had the capability to specifically detect Cu^2+^ and Co^2+^. Additionally, it exhibited excellent characteristics of instantaneous response and ultrahigh sensitivity and selectivity, and could effectively detect target ions in actual samples. Moreover, compared with other fluorescence sensors that could detect multiple ions [46,47], AMN had the unique advantage of being able to distinguish between Cu^2+^ and Co^2+^ via adding glutathione (GSH). AMN-Cu^2+^ and AMN-Co^2+^ could be distinguished selectively through the addition of GSH because the fluorescence intensity of AMN-Cu^2+^ could restore in the presence of GSH. The establishment of this fluorescence sensor provided a direction in terms of the development of single-molecule AIE materials towards multi-ion detection.

## 2. Results and Discussion

### 2.1. Aggregation-Induced Emission

**Figure 1:**
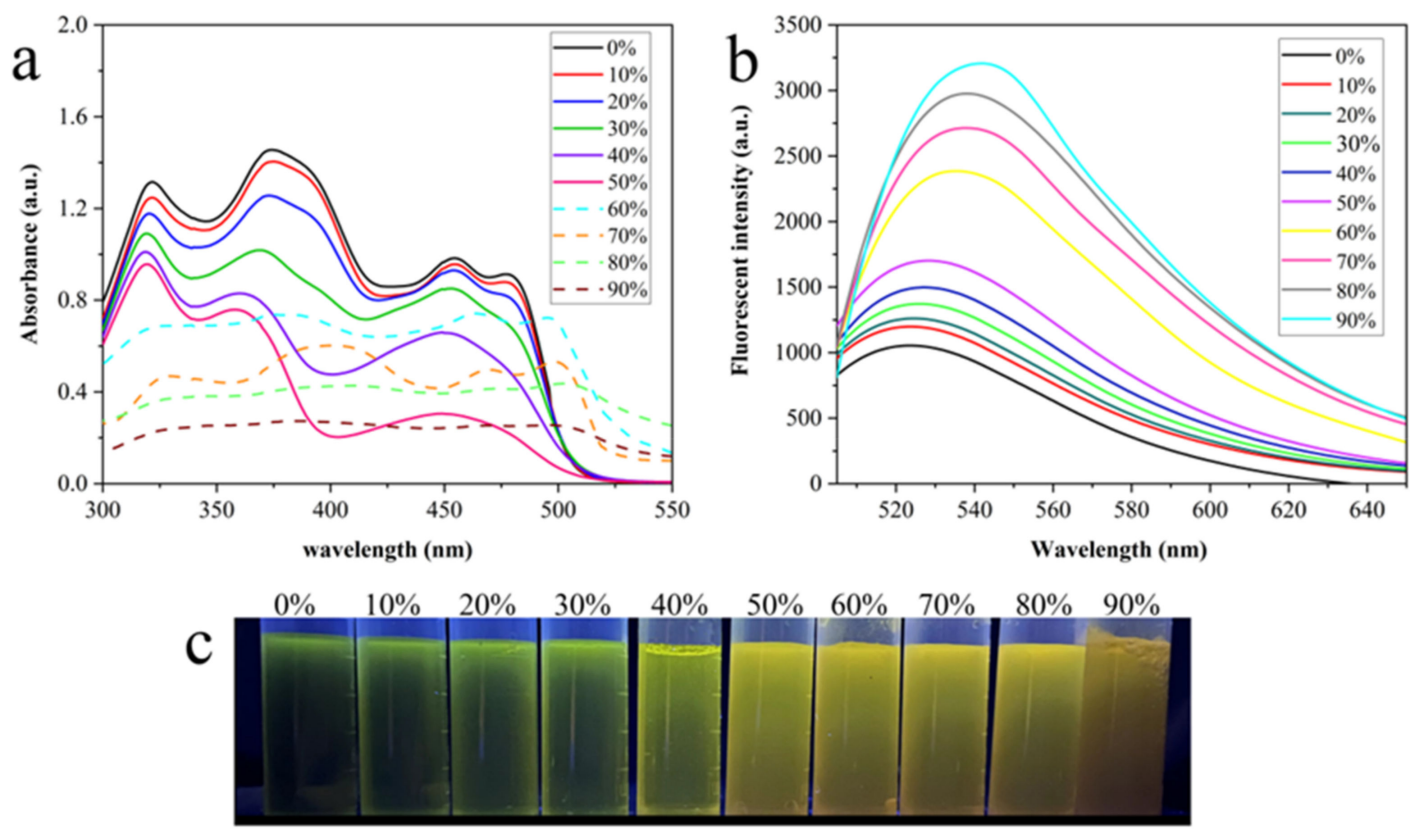
Absorption spectra (**a**) and fluorescence spectra (**b**) of AMN (1 mM) in DMSO/H_2_O mixed solutions at different *f*_w_. (**c**) Photograph of AMN at different *f*_w_ under UV light (365 nm) illumination.

The AIE characteristics of AMN in different water volume fractions (*f*_w_, vol%) were verified. The outcomes of ultraviolet absorption spectra are displayed in Figure 1. In Figure 1a, as the *f*_w_ value raised from 0% to 50%, the UV absorption intensity descended gradually at 370 nm and 450 nm. When *f*_w_ ≥ 60%, both absorption wavelengths redshifted to 400 nm and 470 nm, respectively, which indicated that the dispersed monomolecular probe aggregated accompanied by the increasing of *f*_w_. In Figure 1b, AMN emitted weak fluorescence in the mixtures with low volume fraction of water. As the volume of water increased, the fluorescence strength of AMN gradually increased. When it came to 90%, the fluorescence strength enhanced three times compared with pure DMSO and the emission peak of AMN was redshifted from 528 nm to 540 nm simultaneously. Furthermore, a marked color change in AMN from yellow-green to orange-yellow is observed in Figure 1c, illustrating the AIE property of the compound AMN. In addition, the absolute photoluminescence quantum yield of AMN in solid state was calculated as 7.2%. These results indicate that AMN possesses typical AIE properties.

### 2.2. Characterization of AMN and AMN- Cu^2+^/Co^2+^

**Figure 2:**
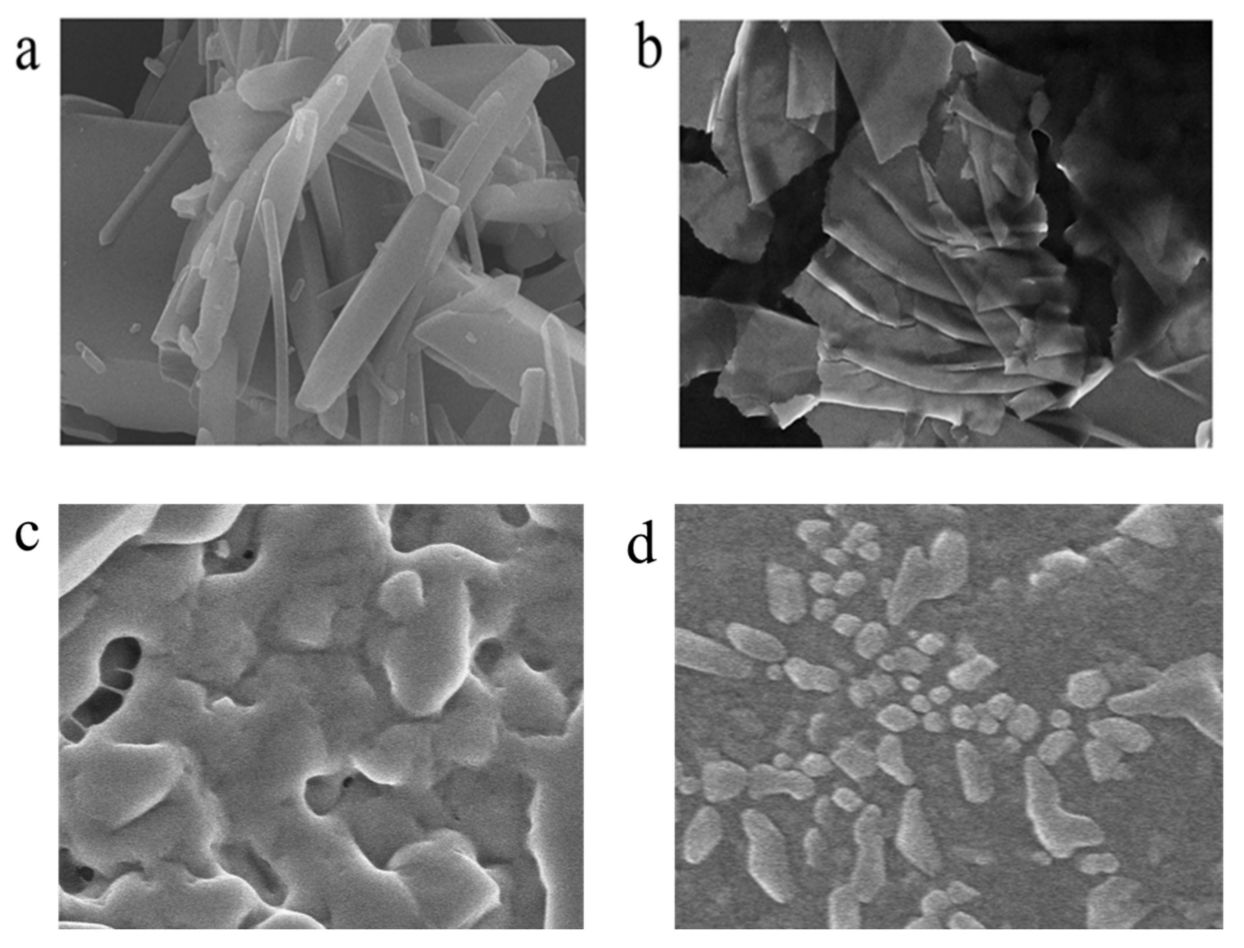
SEM images of AMN in DMSO with 0% (**a**) and 40% (**b**) of water fraction. SEM images of AMN with addition Cu^2+^ (**c**) and Co^2+^ (**d**) in DMSO/H_2_O (*f_w_* = 40%).

The aggregation behavior of AMN in the absence of H_2_O was further studied by SEM in Figure 2. As seen in Figure 2a, AMN presented a virgulate morphology in the DMSO solution (*f*_w_ = 0%). However, when AMN in the DMSO/H_2_O (*f*_w_ = 40%) mixtures, it appeared as a lamellar stacked structure, as seen in Figure 2b, suggesting that the increase in specific surface area was conducive to combining with metal ions. These results demonstrate the typical assembling behavior of AMN in aqueous solution. In addition, as shown in Figure 2c,d, a block-stacking aggregating morphology appeared when Cu^2+^ and Co^2+^ were added. We speculated that the electron-rich N and O atoms in AMN molecular structure interacted with Cu^2+^ and Co^2+^ to form new molecular aggregates. The above results substantiated that the coordination compounds were formed between AMN and Cu^2+^/Co^2+^.

**Figure 3:**
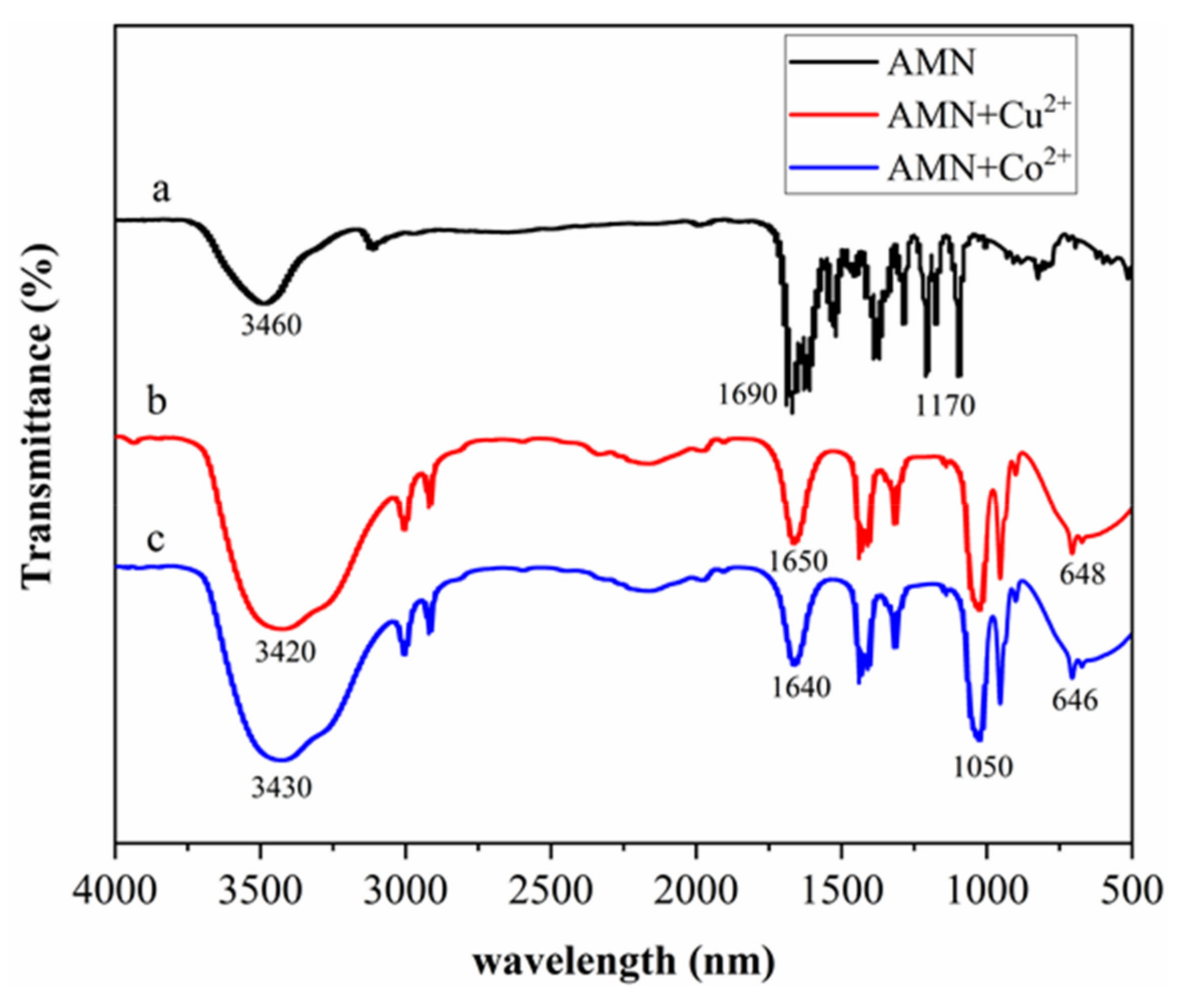
FT-IR spectra of AMN (**a**), AMN-Cu^2+^ (**b**), and AMN-Co^2+^ (**c**).

What can we see in Figure 3 are the FT-IR spectra of AMN (a), AMN-Cu^2+^ (b) and AMN-Co^2+^ (c). In Figure 3a, AMN displays three bands at 3460 cm^−1^,1690 cm^−1^, and 1170 cm^22121^, which could be assigned to stretching vibration peaks of -OH, -C=N, and Ar-O, respectively. The obtained results demonstrate that the Schiff base structure had been formed. The -C=N peaks of AMN combined with Cu^2+^/Co^2+^ were redshifted to 1650 and 1640 cm^−1^, respectively, indicating that the imino nitrogen atom of AMN is involved in the interaction with Cu^2+^ and Co^2+^. Moreover, as shown in Figure 3b,c, the new peaks at 648 cm^−1^ and 646 cm^−1^ can be assigned to vibration peaks of Cu-O and Co-O. At the base of the determination results, the inference that the compound AMN, which sufficed to combine with Cu^2+^ and Co^2+^, was synthesized smoothly.

**Figure 4:**
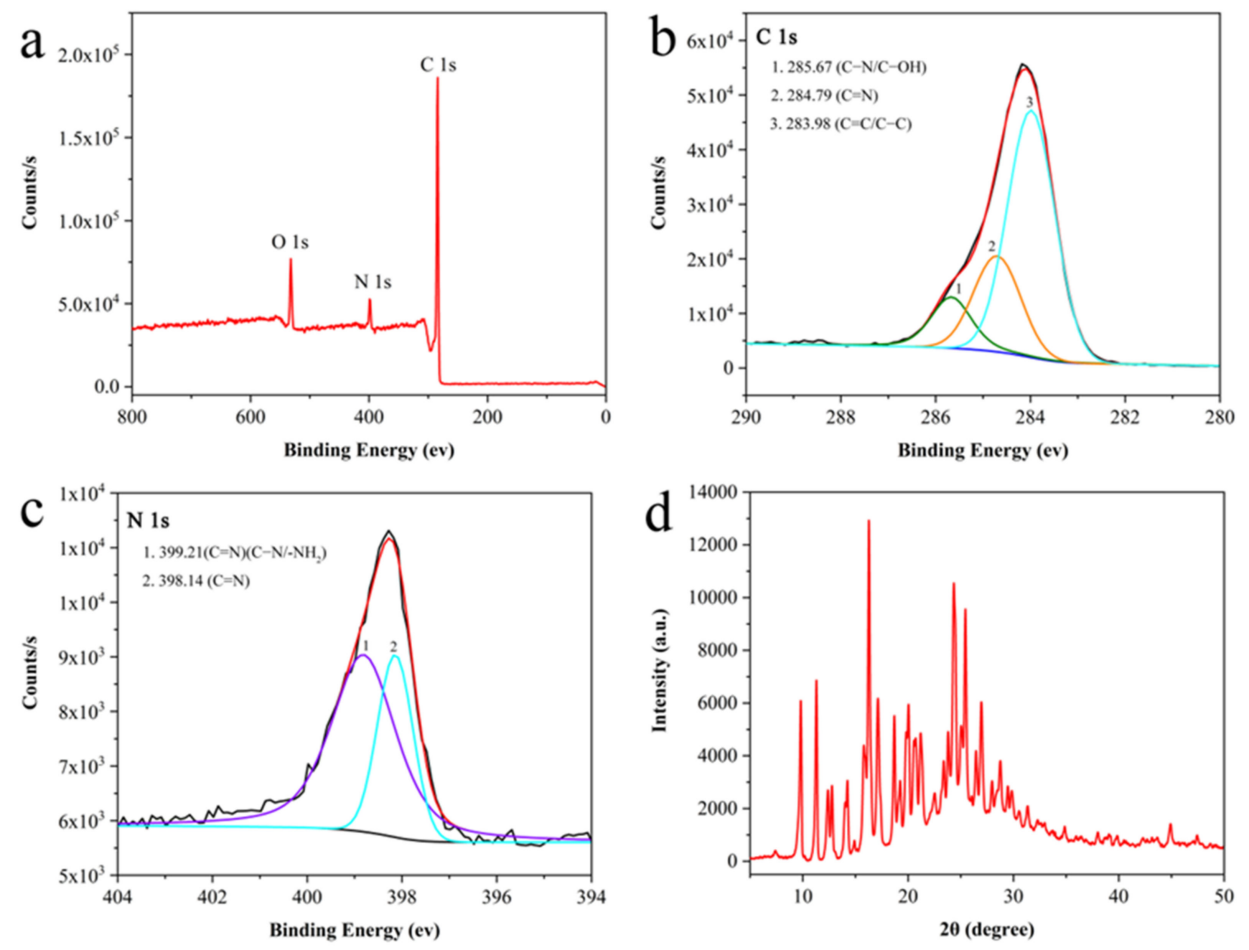
The full-range XPS spectrum of AMN (**a**) and XPS spectrum of C 1s (**b**) and N 1s (**c**). XRD patterns of AMN (**d**).

The chemical composition and crystal structure of AMN were further investigated by XPS and X-ray diffraction analysis (XRD). Figure 4a shows the full-scan XPS spectrum of AMN, demonstrating that the main constituent elements of the AMN are C, N, and O. For the O 1s spectrum, the characteristic peak at 531.9 eV suggested the presence of phenolic hydroxyl groups in the compound. As shown in Figure 4b, the three peaks at 285.67 eV, 284.79, and 283.98 eV constituted the C 1s spectrum of AMN, and they corresponded to the C–N (C–OH) coordination, C=N coordination, and C=C (C–C) coordination, respectively [48]. As illustrated in Figure 4c, the probe displayed characteristic peaks at 399.21 eV and 398.14 eV, which belonged to C=N and C–N (C–NH_2_), respectively. Therefore, the successfully synthesized fluorescent probe had the typical functional group of the Schiff base (C=N). In addition, the XRD patterns of AMN (Figure 4d) showed sharp and intense diffraction peaks, which demonstrated that AMN had a clear crystallite structure.

### 2.3. Optimization of Experimental Conditions

**Figure 5:**
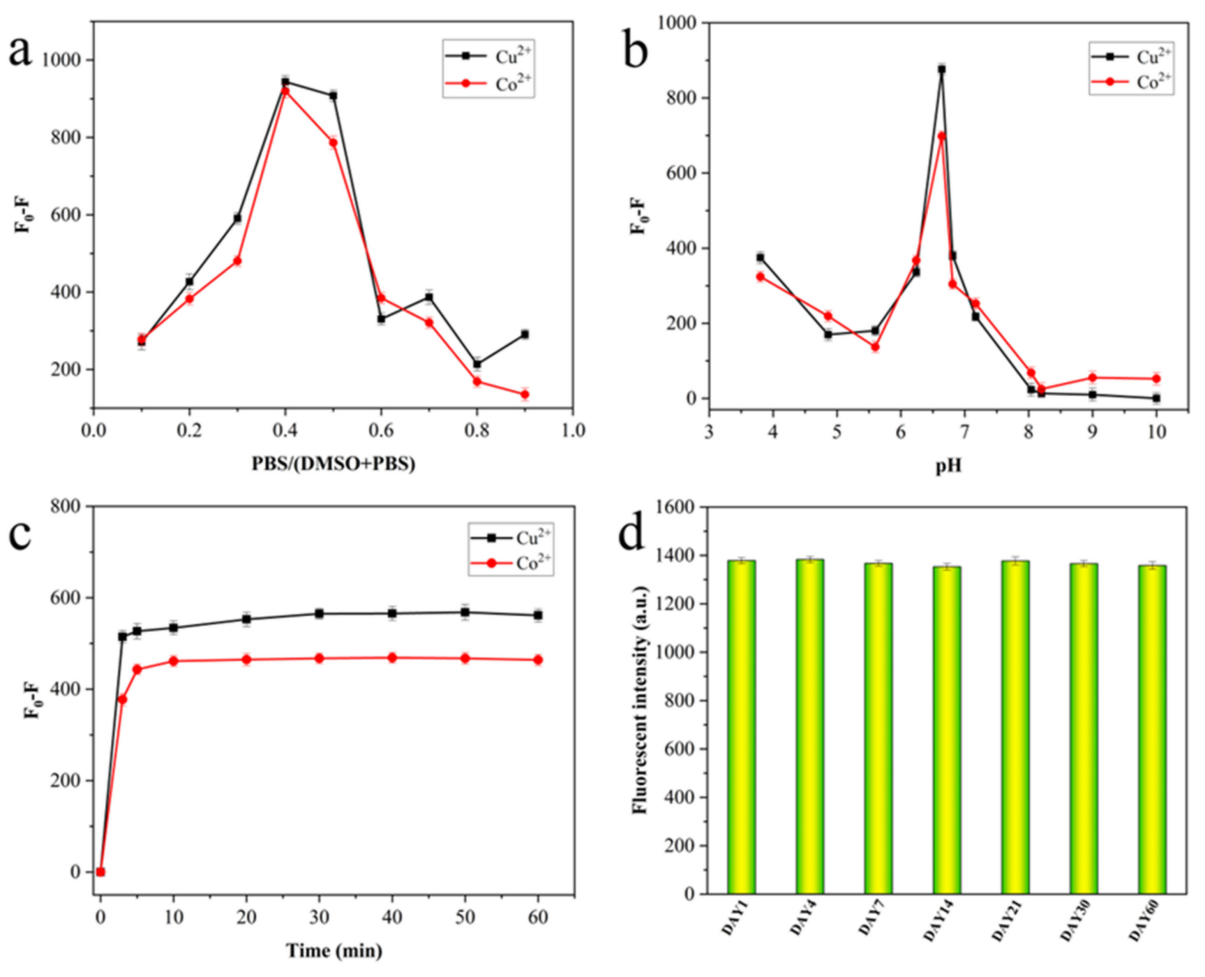
The influence of *f*_w_ (0–90%) (**a**), pH (**b**), and time (**c**) on the fluorescence intensity of AMN in response to Cu^2+^/Co^2+^. Fluorescence intensity of AMN placed for 60 days (**d**).

The optimization of detection condition parameters is the key factor to ensure the sensitivity and effectiveness of fluorescent materials in practical application. Therefore, further experiments were carried out to study the effects of factors such as water volume fraction, pH, and incubation time on the complexation of Cu^2+^ and Co^2+^ by AMN. As shown in Figure 5a, the water volume fraction was in the range of 0–90%, and the difference of fluorescence intensity (F_0_ − F) was the largest when *f*_w_ was 40%, which showed that AMN had a good complexation effect on Cu^2+^ and Co^2+^. Figure 5b shows that the quenching effect of Cu^2+^ and Co^2+^ on AMN was the most obvious when the pH value was 6.8, suggesting that the sensitivity was the maximum at pH 6.8. The effects of the incubation time on the detection of AMN complexed with Cu^2+^ and Co^2+^ in the mixed solution of DMSO/H_2_O (*f*_w_ = 40%, pH 6.8) were investigated by fluorescence spectroscopy. The value of F_0_ − F decreased after the addition of Cu^2+^ and Co^2+^ for 3 min, and stabilized after 5 min (Figure 5c). This result illustrates that the response time of AMN to Cu^2+^ and Co^2+^ was very fast, and it could detect trace amounts of Cu^2+^ and Co^2+^ in real samples in real time. Moreover, this experiment also investigated the change in fluorescence intensity within 60 days of material placement. As shown in Figure 5d, the fluorescence intensity of AMN did not change significantly within 60 days, which indicated that AMN had strong stability.

### 2.4. Spectral Response of AMN to Cu^2+^ and Co^2+^

**Figure 6:**
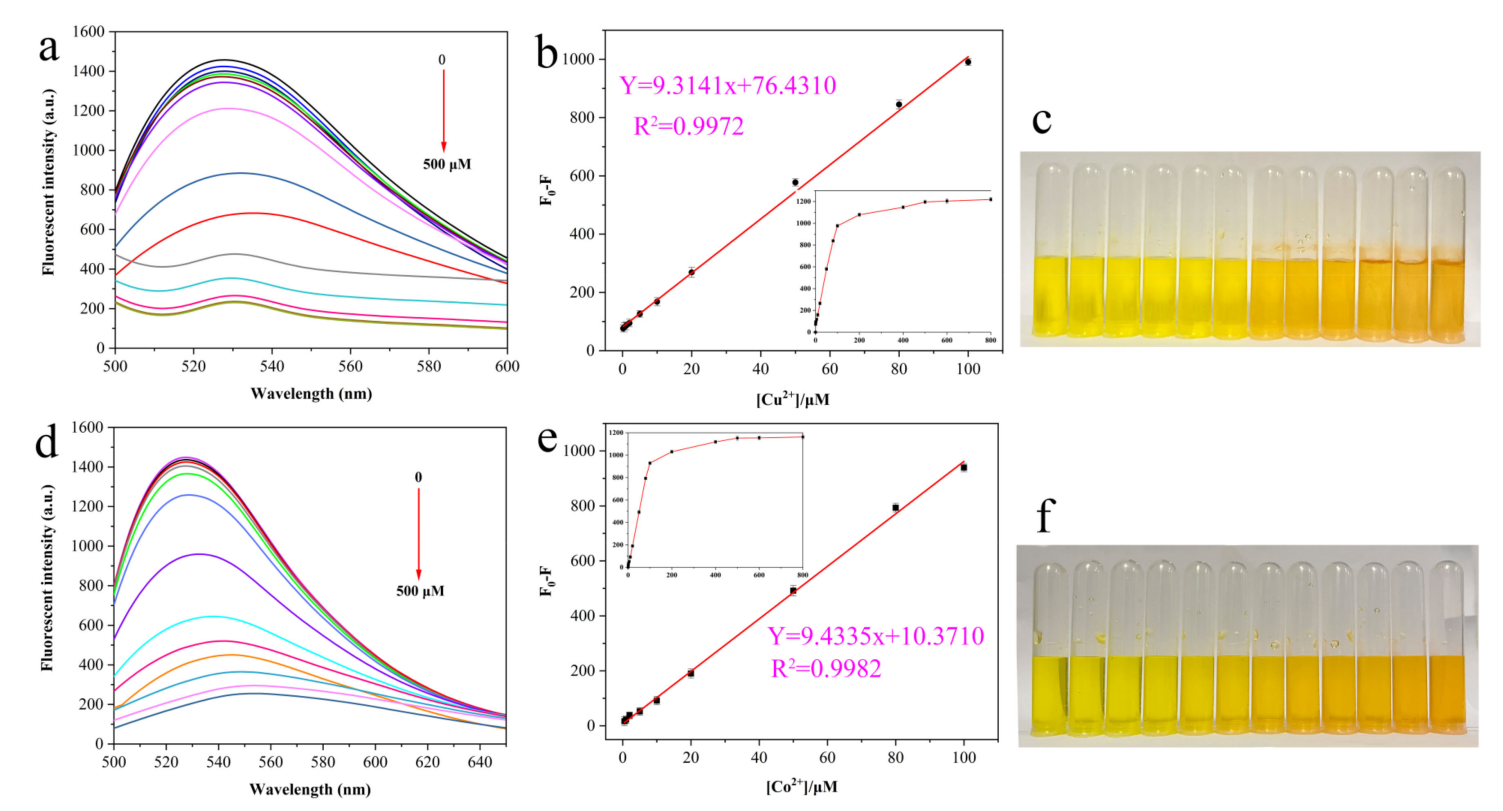
Fluorescence spectra of AMN with different Cu^2+^/Co^2+^ concentrations (**a**,**d**). The difference in fluorescence intensity (F_0_ − F) was linearly related to Cu^2+^/Co^2+^ (**b**,**e**). Inset: Fitting of the AMN fluorescence curve. Color change of AMN in the concentration range of 0–500 μM Cu^2+^/Co^2+^ under sunlight (**c**,**f**).

Under the optimum experiment conditions, fluorescence spectroscopy was applied to further test the responsiveness and sensitivity of AMN to Cu^2+^ and Co^2+^. As displayed in Figure 6a, AMN exhibited satisfactory fluorescence at 528 nm in DMSO/PBS mixed solution, and the fluorescence intensity decreased progressively with the addition of Cu^2+^ increased. In visual observation, the color of the probe AMN gradually deepened with the increase in Cu^2+^ concentration, accompanied by the production of precipitation, suggesting that AMN and Cu^2+^ were complexed with each other (Figure 6c). When the concentration of Cu^2+^ was in the range of 0.2–100 μM, the value of F_0_−F had a good linear relationship with concentration of Cu^2+^, while the correlation coefficient (R^2^) was 0.9972 (Figure 6b) and the LOD was 8.29 × 10^−8^ M based on the formula (1). The fluorescence spectroscopy of AMN in different concentrations of Co^2+^ is presented in Figure 6d. With the increasing addition of Co^2+^, the fluorescence intensity decreased progressively, while the emission peak also red-shifted to 550 nm. Furthermore, the color of AMN gradually deepened in sunlight (Figure 6f). In the range of 0.5–100μM, the relationship between the F_0_−F and the concentration of Co^2+^ was satisfactorily linear, while the R^2^ was 0.9982 (Figure 6e) and the LOD was 9.13 × 10^−8^ M.

**Figure 7:**
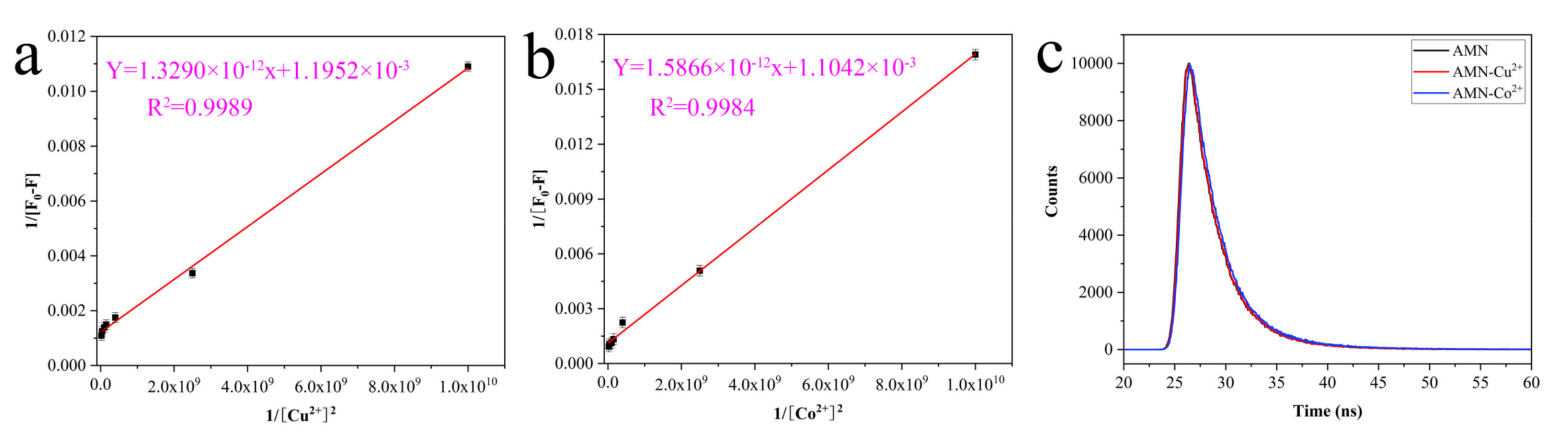
Benesi–Hildebrand plot for calculating the binding K of AMN with Cu^2+^/Co^2+^ from fluorescence results (**a**,**b**). Fluorescence emission decay curve of AMN, AMN-Cu^2+^, and AMN-Co^2+^ (**c**).

According to the Benesi–Hildebrand formula (2), the K of AMN and Cu^2+^ was 8.94 × 10^8^ M^−1^ (Figure 7a); in the same way, the K of AMN and Co^2+^ was 6.96 × 10^7^ M^−1^ (Figure 7b). Meanwhile, in order to study the quenching mechanism, the fluorescence decay curves were fitted toward AMN, AMN-Cu^2+^, and AMN-Co^2+^ (Figure 7c), and the average fluorescence lifetime was obtained by second-order fitting. The average lifetime of AMN (3.11 ns) was approximately the same as those AMN-Cu^2+^ (3.01 ns) and AMN-Co^2+^ (3.10 ns), suggesting that the decrease in fluorescence intensity of AMN caused by Cu^2+^ and Co^2+^ could be ascribed to the static quenching process [49]. Although a variety of fluorescence chemical sensors were reported for monitoring Cu^2+^ or Co^2+^, they suffered from low sensitivity in application, so most of the probes with a single signal output could only detect an analyte. Compared with the reported fluorescence chemosensors, AMN was equipped with higher sensitivity and broader application prospects, as seen in Table 1.

### 2.5. Selectivity and Interference

**Figure 8:**
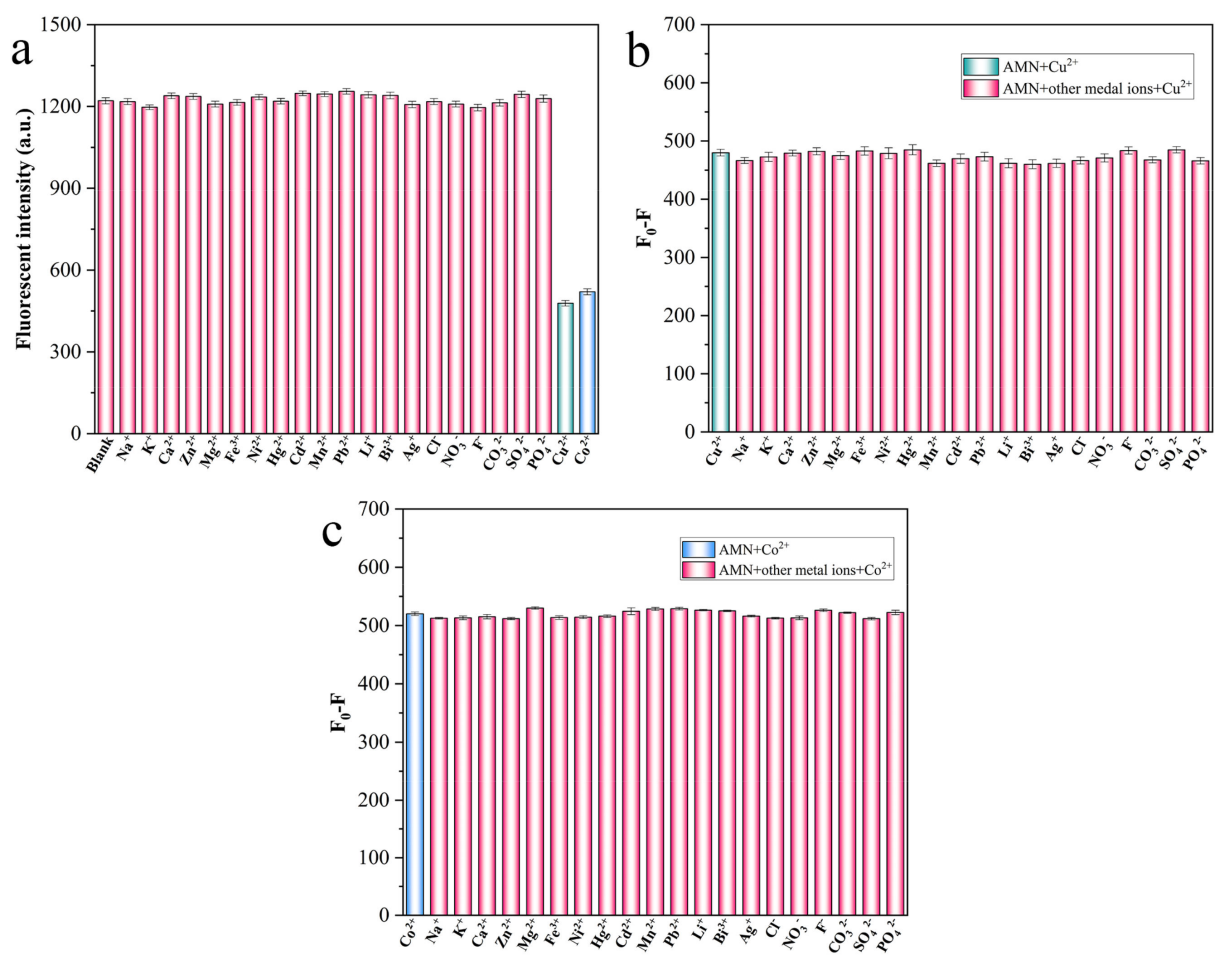
Fluorescence intensity of AMN in other ions (0.5 mM) and Cu^2+^/Co^2+^ (0.1 mM) (**a**). Fluorescence intensity of AMN in the coexistence of other ions and Cu^2+^/Co^2+^ (**b**,**c**).

Fluorescence responses of AMN in DMSO/PBS (*v*/*v* = 3:2) mixed solution to diverse anions and metal cations were evaluated, aiming to investigate the fluorescence sensitivity of AMN to Cu^2+^ and Co^2+^. As was evident from the results in Figure 8a, at the presence of common anions (Cl^−^, NO_3_^−^, F^−^, CO_3_^2−^, SO_4_^2−^ and PO_4_^2−^) and cations (Na^+^, K^+^, Ca^2+^, Zn^2+^, Mg^2+^, Fe^3+^, Ni^2+^, Hg^2+^, Cd^2+^, Mn^2+^, Pb^2+^, Li^2+^, Bi^+^ and Ag^+^), the fluorescence intensity of AMN only significantly expressed the trend of decrease to Cu^2+^ and Co^2+^, indicating the high sensitivity of AMN to Cu^2+^ and Co^2+^.

Additionally, to study the detectability of AMN to Cu^2+^ and Co^2+^ in complicated circumstances, an anti-interference experiment was implemented. The obtained results showed that the fluorescence responsiveness of AMN to Cu^2+^ and Co^2+^ was satisfactory in the complicated background that other competing ions presented (Figure 8b,c). In summary, AMN revealed benign sensitivity and specificity to Cu^2+^ and Co^2+^.

### 2.6. Binding Mechanism Studies of Probe AMN to Cu^2+^ and Co^2+^

**Figure 9:**
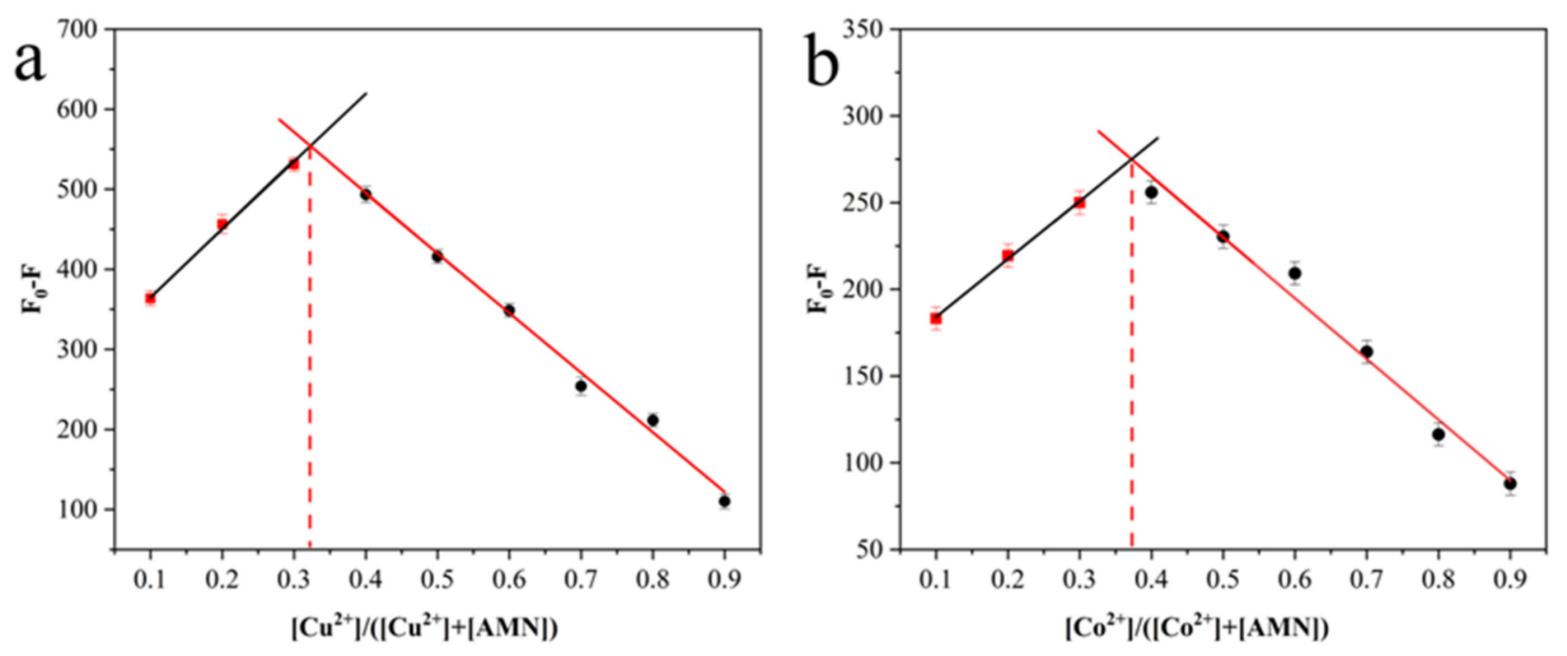
Job’s plot for determination of stoichiometry of AMN-Cu^2+^ (**a**) and AMN-Co^2+^ (**b**) complex. The total concentration of AMN and Cu^2+^/Co^2+^ was 1 mM.

In consideration of the results achieved anteriorly, the binding of AMN to Cu^2+^ and Co^2+^ was notarized in 2:1 stoichiometric proportion. Furthermore, in order to futher understand the binding stoichiometry of AMN and Cu^2+^/Co^2+^ more precisely, itwas proved by means of Job’s plot. The experimental procedures that change the mole fraction of Cu^2+^/Co^2+^ under the same overall concentration of AMN and Cu^2+^/Co^2+^ were taken to record the fluorescence intensity. It can be seen from Figure 9a, the fluorescence quenching effect was the most significant when the value of [Cu^2+^]/([Cu^2+^]+[AMN]) reached 0.33, indicating that the binding of AMN to Cu2+ was in 2:1 stoichiometric proportion; in the same way, when the value of [Co^2+^]/([Co^2+^] + [AMN]) reached 0.37, the fluorescence quenching effect was the most significant, so the binding of AMN to Co^2+^ was in 2:1 stoichiometric proportion (Figure 9b). The results coincide exactly with those of the fluorescence linear tests [56].

**Figure 10:**
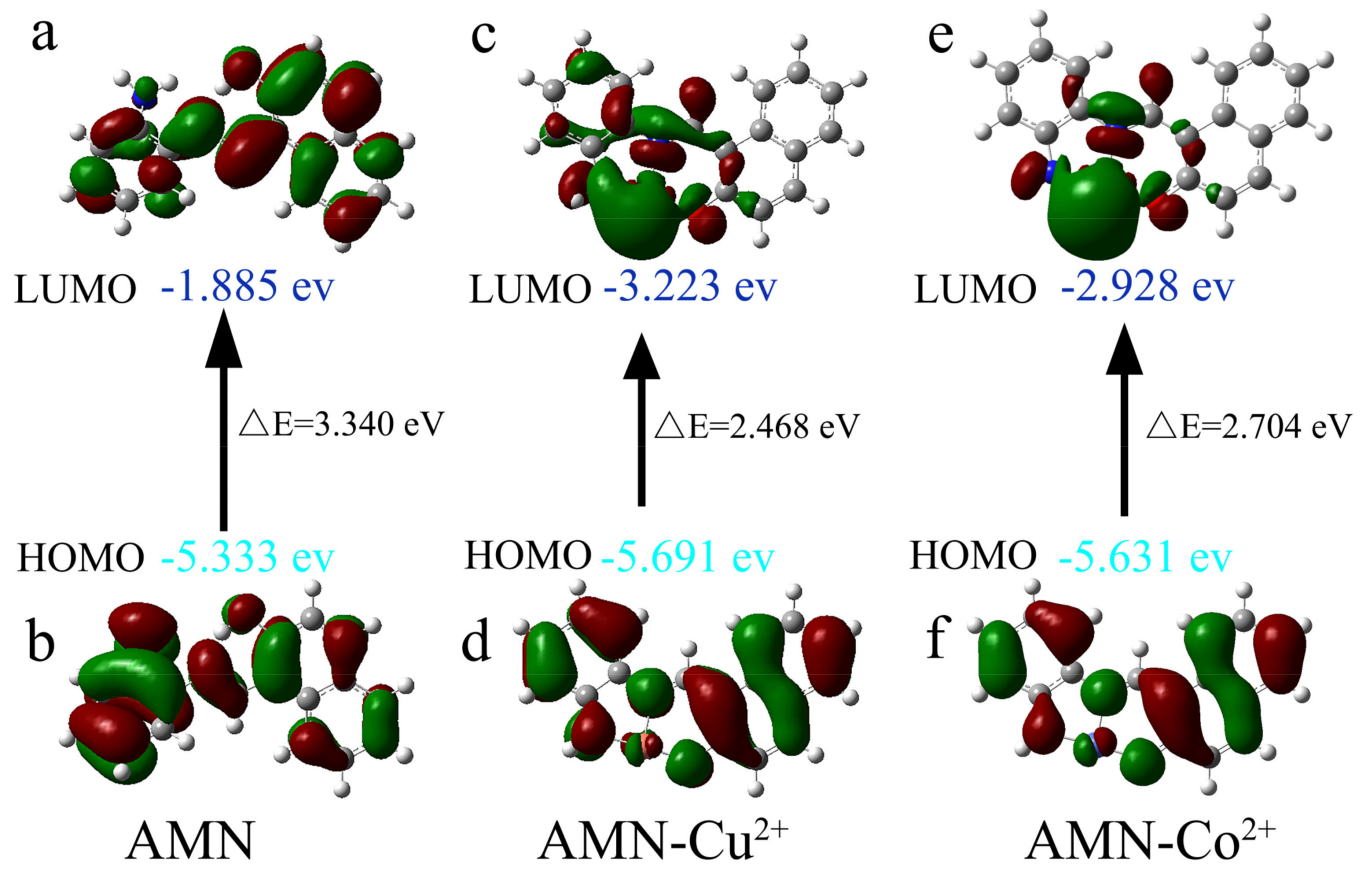
HOMO-LUMO energy level diagram of AMN and its corresponding Cu^2+^/Co^2+^ complex (**a**–**f**).

To know more about the geometrical and electric structures between AMN and its complexes, DFT was put into experiment. According to the binding stoichiometry of AMN and its complexes, the spatial distribution and orbital energies of the highest occupied molecular orbital (HOMO) and the lowest unoccupied molecular orbital (LUMO) were calculated and are presented in Figure 10. As shown in Figure 10a–f, when metal ions coordinated with AMN, the energy level difference (ΔE) between the HOMO and LUMO of AMN-Cu^2+^ (2.468 eV) and AMN-Co^2+^ (2.704 eV) were lower than the value of AMN (3.448 eV) between HOMO and LUMO. The value of the ΔE was smaller, the intensity of fluorescence was lower, and the decrease in the ΔE corresponding to the change in intramolecular charge transfer (ICT) was attributed to the complexation of AMN to Cu^2+^ and Co^2+^. Moreover, the LUMO electron density of AMN was distributed throughout the molecule, while the LUMO electron density of AMN-Cu^2+^ and AMN-Co^2+^ was principally distributed around the metal, which also confirmed that complexation of AMN to Cu^2+^ and Co^2+^ restrained the excited-state intramolecular proton transfer (ESIPT) and activated ICT, forming a new conjugated structure around the metal ion. The results above all verified the presence of Cu^2+^ and Co^2+^, resulting in the fluorescence quenching effect of AMN.

**Figure 11:**
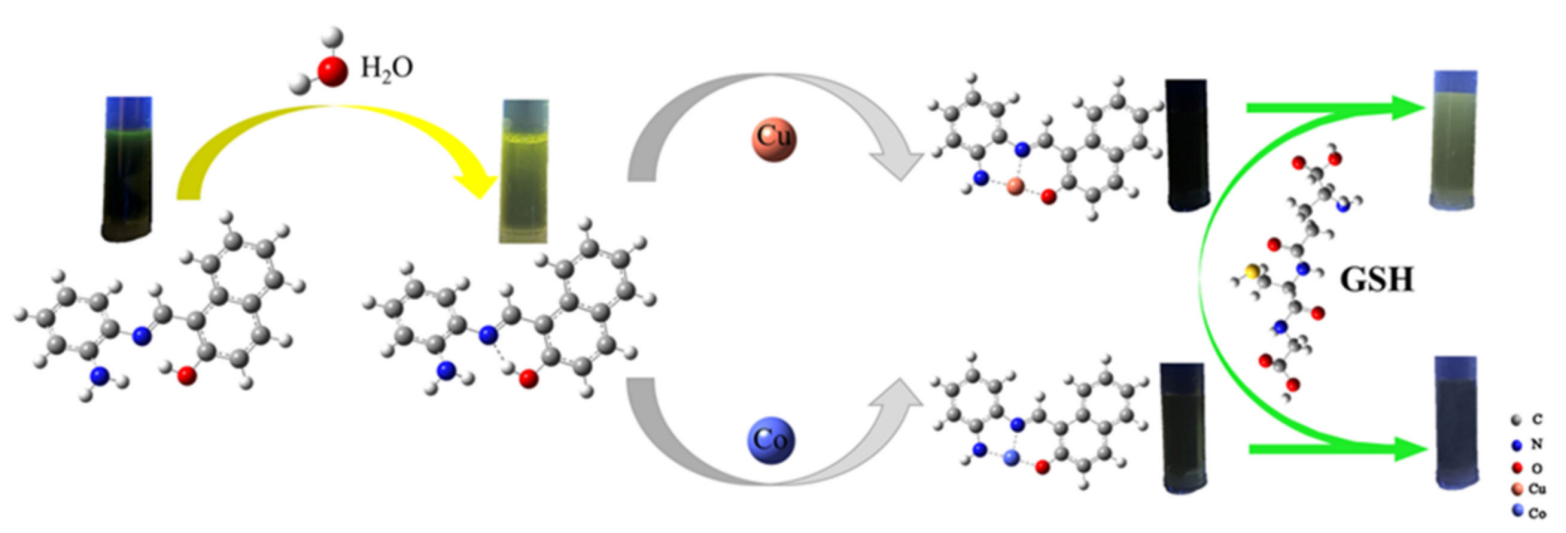
Mechanism diagram of AMN detection of Cu^2+^ and Co^2+^.

The mechanism of the complexation of AMN to Cu^2+^/Co^2+^ is shown in Figure 11; AMN in DMSO (*f_w_* = 0%) solution could be rotated optionally via the C-C bond and C=N bond, generating the nonradiative decay and feeble fluorescence emission [57]. Furthermore, the increase in the solution of *f*_w_ promoted the ESIPT and limited the internal rotation, leading to quick increase in fluorescence intensity and redshift of the emission wavelength [58]. After further addition Cu^2+^/Co^2+^ to the mixed solution of DMSO/PBS (*v*/*v* = 3:2), the fluorescence intensity decreased, which inhibited the ESIPT process and helped to produce charge transfer, resulting in the fluorescence quenching effect being generated.

### 2.7. Distinction between Cu^2+^ and Co^2+^ by GSH

**Figure 12:**
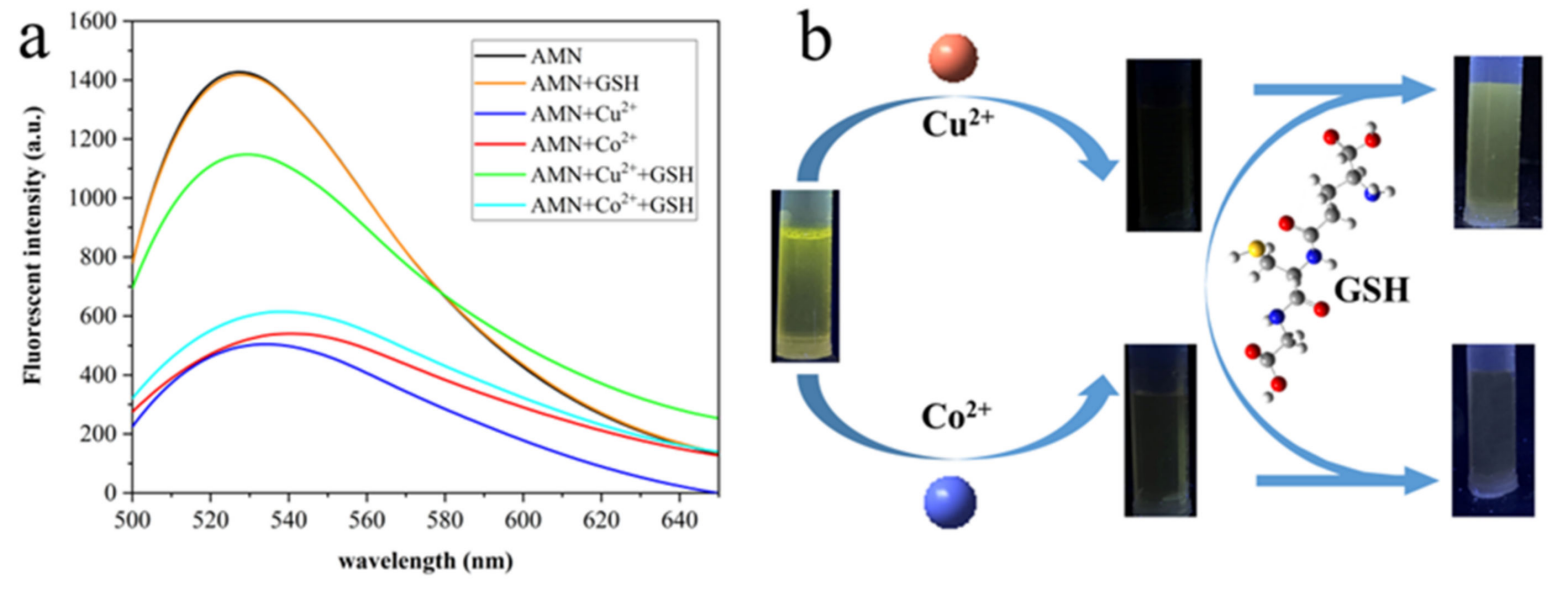
Fluorescence intensity of AMN-Cu^2+^ and AMN-Co^2+^ in the presence and absence of GSH (**a**). Fluorescence intensity of AMN-Cu^2+^ and AMN-Co^2+^ under 365 nm UV lamp after adding GSH (**b**).

It is well known that the Cu^2+^ displacement method is a special and effective fluorescence detection method for thiol-containing (-SH) molecules [59]. There are three common thiol compounds, namely cysteine, homocysteine, and GSH. In this experiment, GSH was selected to distinguish Cu^2+^ from Co^2+^. As shown in Figure 12, upon the incremental addition of five equivalents of GSH (500 μM), the fluorescence intensity of AMN-Cu^2+^ increased by about 3 times at 528 nm. Under UV light (365 nm) illumination, a remarkable color change from colorless to green was observed, and the fluorescence intensity was restored. However, the fluorescence intensity and color did not change significantly in the AMN-Co^2+^ system. These differences could be attributed to the fact that the -SH carried by GSH could combine Cu^2+^ in the AMN-Cu^2+^ system and generate free AMN, but it is not easy to combine Co^2+^ in the AMN-Co^2+^ system [60]. Therefore, AMN was conducted to independently recognize Cu^2+^ and Co^2+^ in the case of GSH, which could provide a new design idea for the detection of multiple ions.

### 2.8. Actual Sample Testing

The potential ability of probe AMN was investigated to the determination of Cu^2+^ and Co^2+^ in tap water, river water, and yellow croaker. The detection accuracy and relative standard deviations (RSDs) were studied by adding different concentrations of Cu^2+^ and Co^2+^ to the samples and calculating recoveries. As shown in Tables S1 and S2, the value of recoveries of Cu^2+^ and Co^2+^ in the actual samples were found from 98.61% to 103.27%, and the RSDs were less than 2.55%, indicating that the method could accurately determine the content of Cu^2+^ and Co^2+^ in the environment and food and had a good application prospects.

## 3. Materials and Methods

### 3.1. Chemicals and Reagents

All chemicals and solvents were supplied by commercial suppliers and could be used directly without further purification. One of the raw materials, 2-hydroxy-1-naphthaldehyde and glutathione (GSH), were obtained from Bide Pharmaceutical Technology Co., Ltd. (Shanghai, China). The other O-phenylenediamine (OPD) was acquired from Shanghai Yien Chemical Technology Co., Ltd. (Shanghai, China). NaCl, KCl, CaCl_2_, K_2_SO_4_, Na_2_CO_3_, Mg(NO_3_)_2_•2H_2_O were supplied by Hengxing Chemical Reagent Co., Ltd. (Tianjin, China). CdCl_2_ LiBr, FeCl_3_, AgNO_3_ and ZnCl_2_ were offered by Tianjin Damao Chemical Reagent (Tianjin, China). MnCl_2_, CoCl_2_•6H_2_O, Ni_2_SO_4_, PbCl_2_, HgSO_4_, BiCl_3_, and CuCl_2_ were provided by Shanghai Macklin Biochemical Co., Ltd. (Shanghai, China).

### 3.2. Instruments

Fluorescence detections were carried out on F380 fluorescence spectrometer (Tianjin Port East Technology Co., Ltd., Tianjin, China). Fourier transform infrared spectroscopy (FT-IR) spectra were executed on a Nicolet 6700 FT-IR spectrometer (Thermo Fisher Co., Waltham, MA, USA). The absorbance was measured by TU-1810 UV-visible spectrophotometer from Beijing Puyang General Instrument Co., Ltd. (Beijing Puyang General Instrument Co., Ltd., Beijing, China) The surface distribution information and chemical states of element were gained by Thermo Fisher EscaLab 250Xi X-ray photoelectron spectroscopy (XPS). The surface structure was analyzed by JEOL JSM-4800 scanning electron microscope. Mass spectra were obtained on the Bruker-Micro TOF-QII mass spectrometer (Bruker Co., Ltd., Karlsruhe, Germany). The pH value was recorded using a PH-3W pH meter (INESA Scientific Instruments Co., Ltd., Shanghai, China). ^1^H NMR and ^13^C NMR spectra were performed on Bruker Avance NEO using methanol deuteride as solvent at room temperature (Bruker Co., Ltd., Karlsruhe, Germany).

### 3.3. Synthesis of AMN

**Scheme 1:**
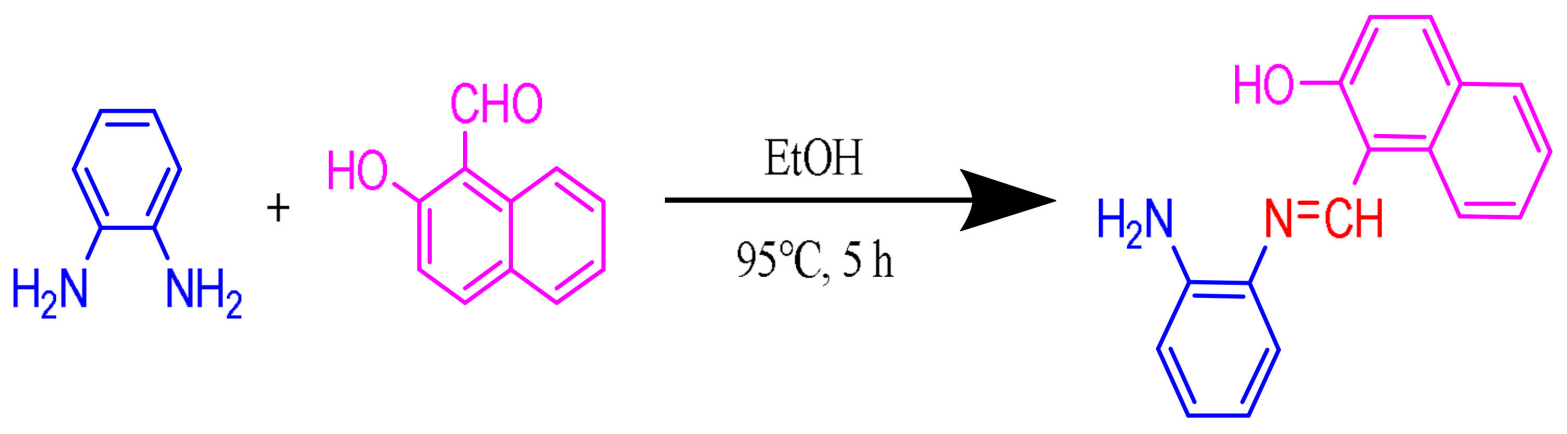
The synthesis routing of AMN.

The synthetic route for AMN was shown in Scheme 1. O-Phenylenediamine (0.27 g, 2.5 mM) was dissolved in ethanol (10 mL) in a round-bottom flask, then 20 mL anhydrous ethanol solution of 2-hydroxy-1-naphthaldehyde (0.86 g, 5 mM) was gradually added. The mixture was refluxed accompanied by 3 drops of glacial acetic acid and stirred at 95 °C for 5 h. The reaction was slowly cooled to room temperature. Subsequently, the resulting compound was obtained by filtration and carefully washed with ethanol. Ultimately, the yellow precipitate AMN was yielded through drying under vacuum (yield: 88.55%, 0.58 g). ^1^H NMR (400 MHz, (Methyl sulfoxide)-d6) δ (ppm): 15.16 (s, 1 H), 9.68 (s, 1 H), 8.54 (d, J = 8.5 Hz, 1 H), 7.95 (d, J = 9.1 Hz, 1 H), 7.84–7.82 (m, 1 H), 7.82–7.79 (d, J = 9.1 Hz, 1 H), 7.56–7.53 (m, 1 H), 7.45–7.43 (m, 1 H), 7.38–7.35 (m, 1 H), 7.07 (d, J = 9.1 Hz, 1 H) (Figure S1). ^13^C NMR (100 MHz, (Methyl sulfoxide)-d6) δ (ppm): 169.12, 157.75, 138.94, 137.24, 133.50, 129.46, 128.61, 127.84, 127.35, 124.07, 121.97, 121.07, 120.15, 109.73 (Figure S2). ESI-MS *m/z*: Calculated for C_17_H_14_N_2_O [M + H]^+^: 262.1106, found: 262.1167 (Figure S3).

### 3.4. Spectral Measurement

AMN (1 mM) dissolved in DMSO was prepared as a stock solution and stored at 4 °C for further use. In addition, all stock solutions (1 mM) of various metal cations were prepared for spectrometry after being diluted appropriately. At room temperature, all experimental samples for UV-Vis and fluorescence were allowed to be measured in DMSO/PBS (5 mL, *v*/*v* = 3:2) buffer solution. Each experiment was performed more than three times in order to obtain reliable and accurate data.

### 3.5. Determination of the Detection of Limit (LOD) and Association Constant (K)

The LOD of AMN for Cu^2+^ and Co^2+^ were determined by fluorescence spectroscopy and calculated by the following equation:

LOD = 3σ/k(1)

where σ is the standard deviation of the blank sample and k represents the slope of the changes in fluorescence intensity (F_0_ − F) versus to the concentration of Cu^2+^ and Co^2+^.

Upon the 2:1 stoichiometry between AMN and guest cation (Cu^2+^ or Co^2+^), the below Benesi–Hildebrand equation was used to calculate the association constant K [61]:

(2) $$\frac{1}{F-F_{0}}=\frac{1}{F^{\prime }-F_{0}}+\frac{1}{{K(F}^{\prime }-F_{0}){\left[M\right]}^{2}}$$

where F_0_ and F represent the fluorescence emission intensity of AMN at 528 nm in the absence and the presence of targeted metal ions (Cu^2+^ or Co^2+^). F′ is the fluorescence intensity of the fully complexed form when the added Cu^2+^ or Co^2+^ concentration reaches the maximum, and K is the association constant.

### 3.6. Calculation of Density Functional Theory

All computational studies were performed by density functional theory (DFT), Becke’s three-parameter nonlocal exchange function and Lee–Yang–Parr nonlocal correlation function (B3LYP) in the Gauss 09 program [62].

### 3.7. Analysis of Real Samples

To evaluate the potential application of AMN for determining Cu^2+^ and Co^2+^, their three concentrations were subjected to adding to three samples containing tap water, yellow croaker, and river water. The tap water was from our campus, the yellow croaker was purchased from a supermarket in Shenyang, and the river water was collected from Xihu District, Benxi. The specific steps of the pretreatment of small yellow croaker were as follows: freeze-dried small yellow croaker powder (0.2 g) and nitric acid (5 mL) were mixed in an autoclave, which was placed in an oven at 180 °C for 90 min. Then, the mixtures in the autoclave were moved to the fume hood to cool to room temperature. Subsequently, the digestion solution was transferred to a volumetric flask and diluted with PBS buffer (pH 6.8) for further detection.

## 4. Conclusions

In conclusion, we demonstrated the use of a new fluorescent probe AMN, which could act as a chemical sensor for the dual detection of Cu^2+^ and Co^2+^ with excellent selectivity and high sensitivity. Moreover, AMN displayed outstanding AIE properties, which could be served for rapid “naked eye” detection based on obvious color changes. The LODs of AMN to Cu^2+^ and Co^2+^ were calculated as 8.29 × 10^−^^8^ M and 9.13 × 10^−^^8^ M. The binding ratios of AMN and metal ions were proven to be 2:1 by the Jobs’ plot method. According to DFT calculations and other characterizations, the quenching mechanism of metal-complexed AMN was further confirmed. Furthermore, a distinction was successfully achieved between AMN-Cu^2+^ and AMN-Co^2+^ based on the different “on” and “off” fluorescence performance under excessive GSH. On the basis of the above studies, this efficient bifunctional chemosensor platform based on the “on–off” fluorescence detection strategy was established, which provided significant guidance for the development of single-molecule sensors for multiple ion detection.

## Figures and Tables

**Table 1:** Comparison of the presented method with reported methods.

Materials	Response Ion	Test Media	LOD (μM)	Ref.
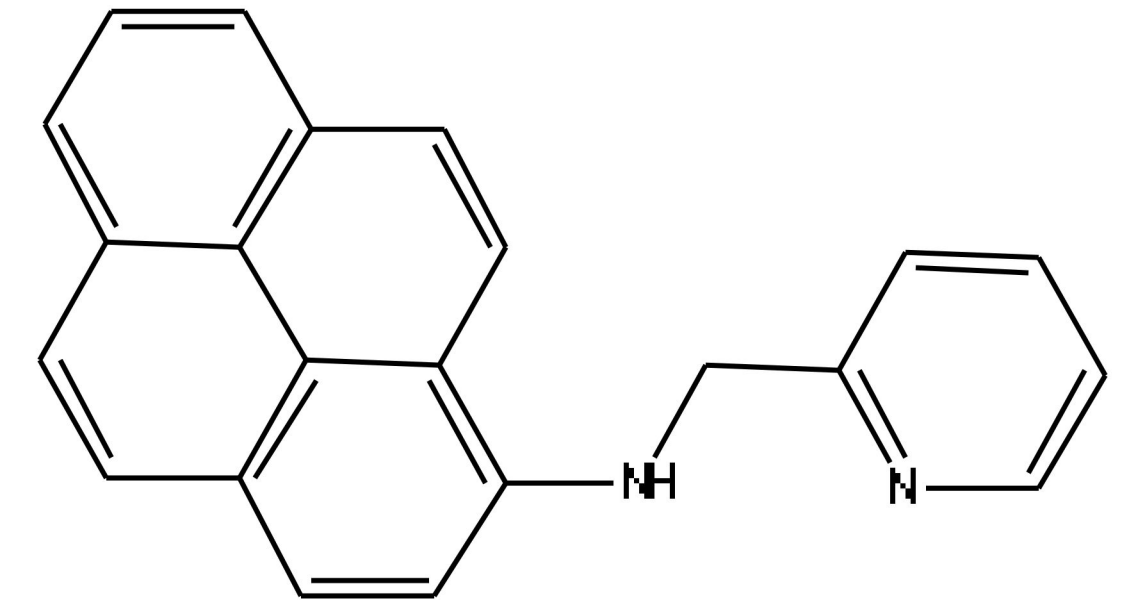	Cu^2+^	CH_3_CN/H_2_O (1:1)	0.19	[50]
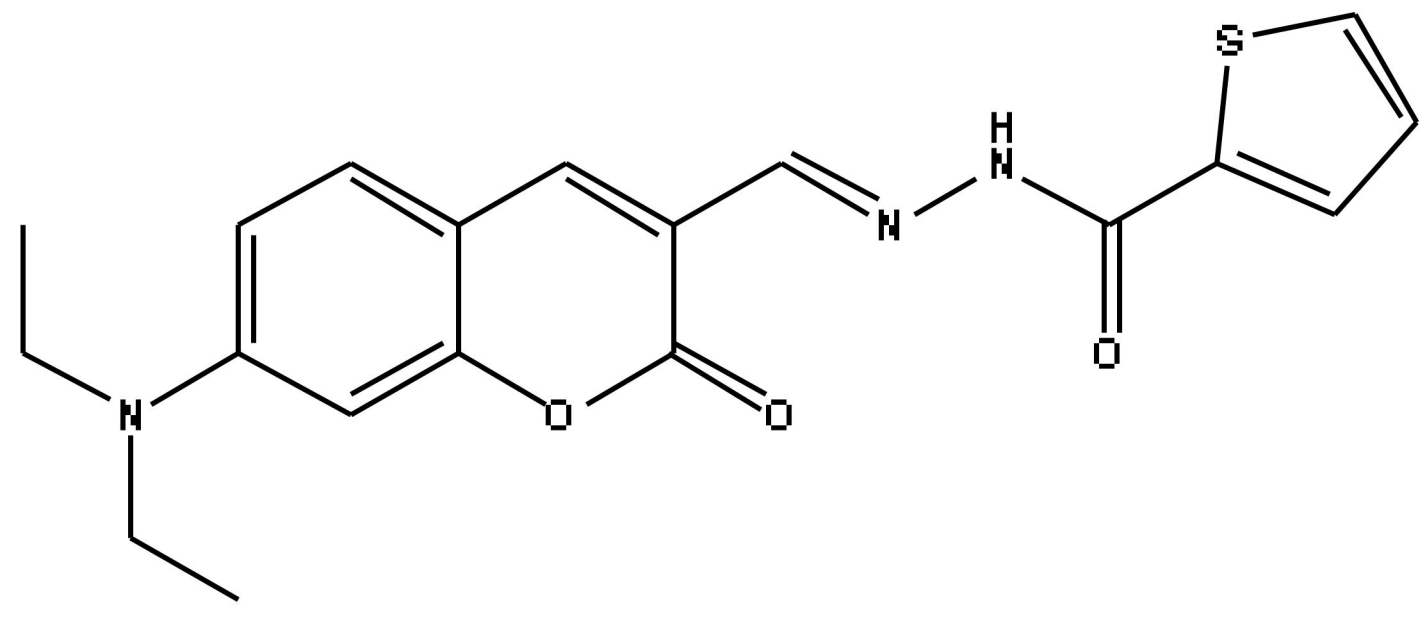	Cu^2+^	EtOH/H_2_O (1:99)	0.14	[51]
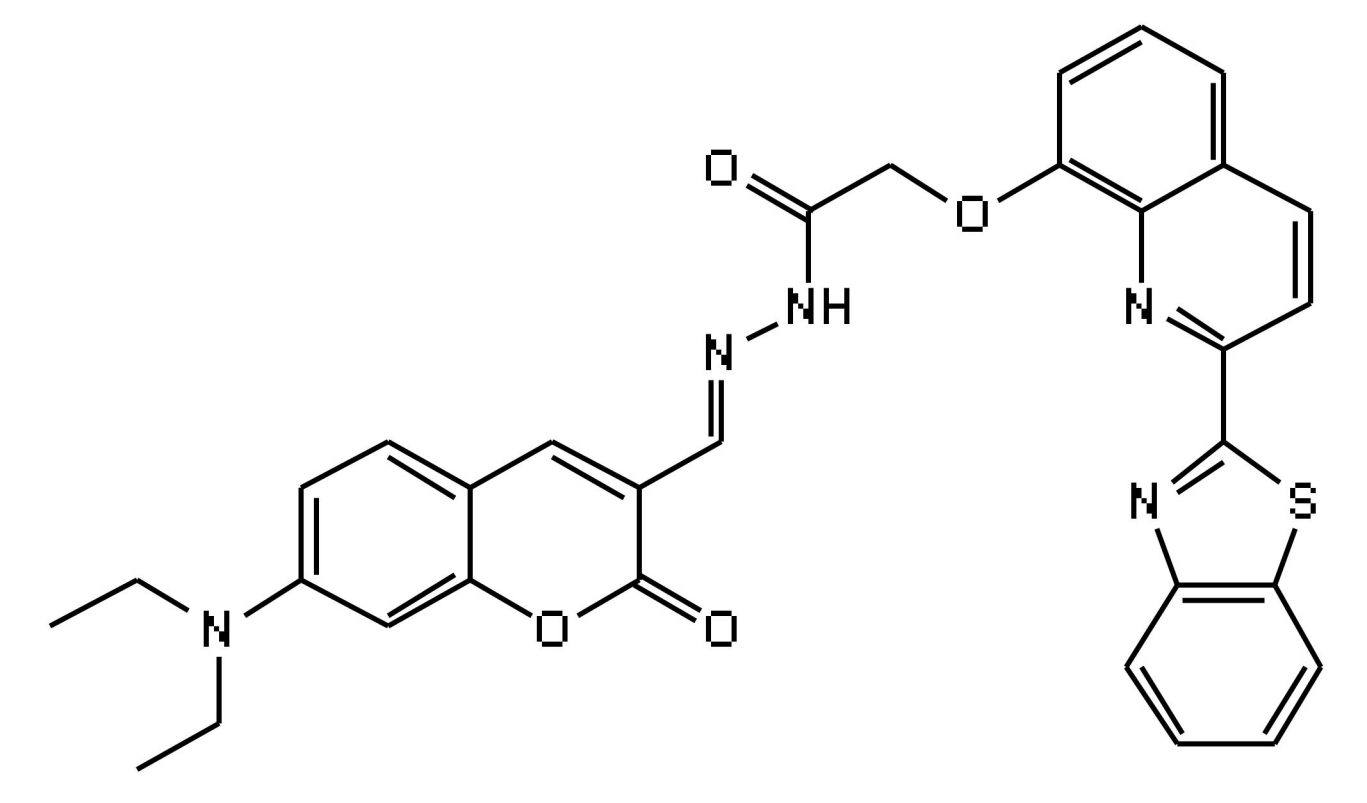	Cu^2+^	DMSO/HEPES (3:2)	0.060	[52]
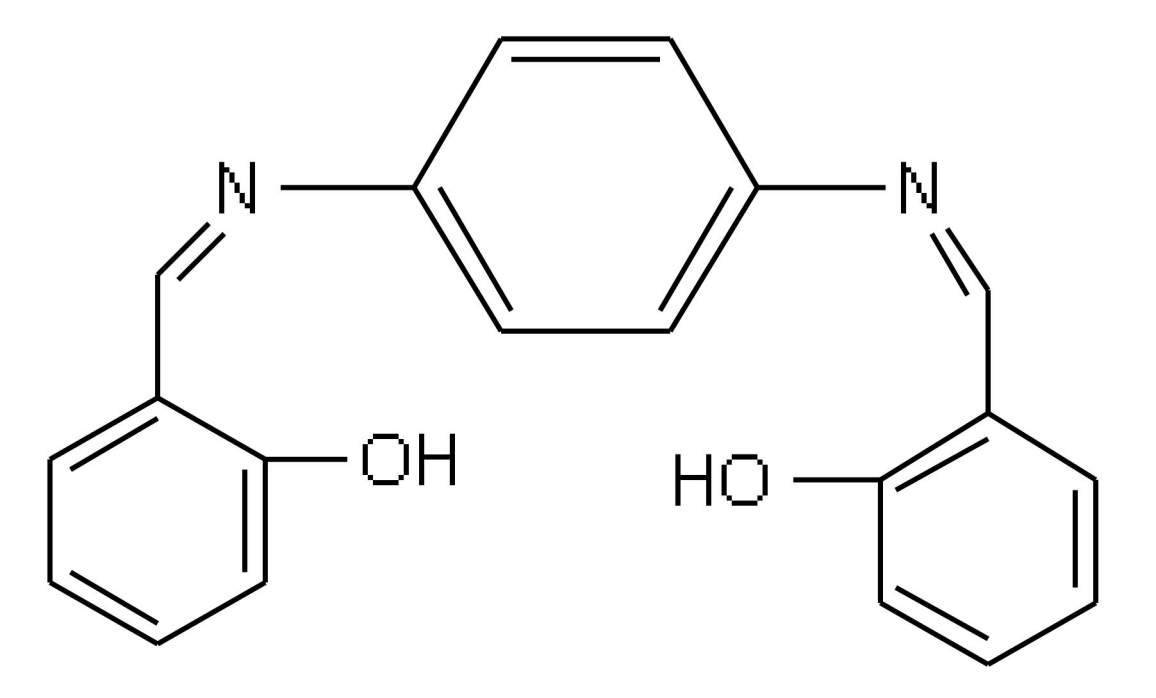	Co^2+^	EtOH	0.78	[31]
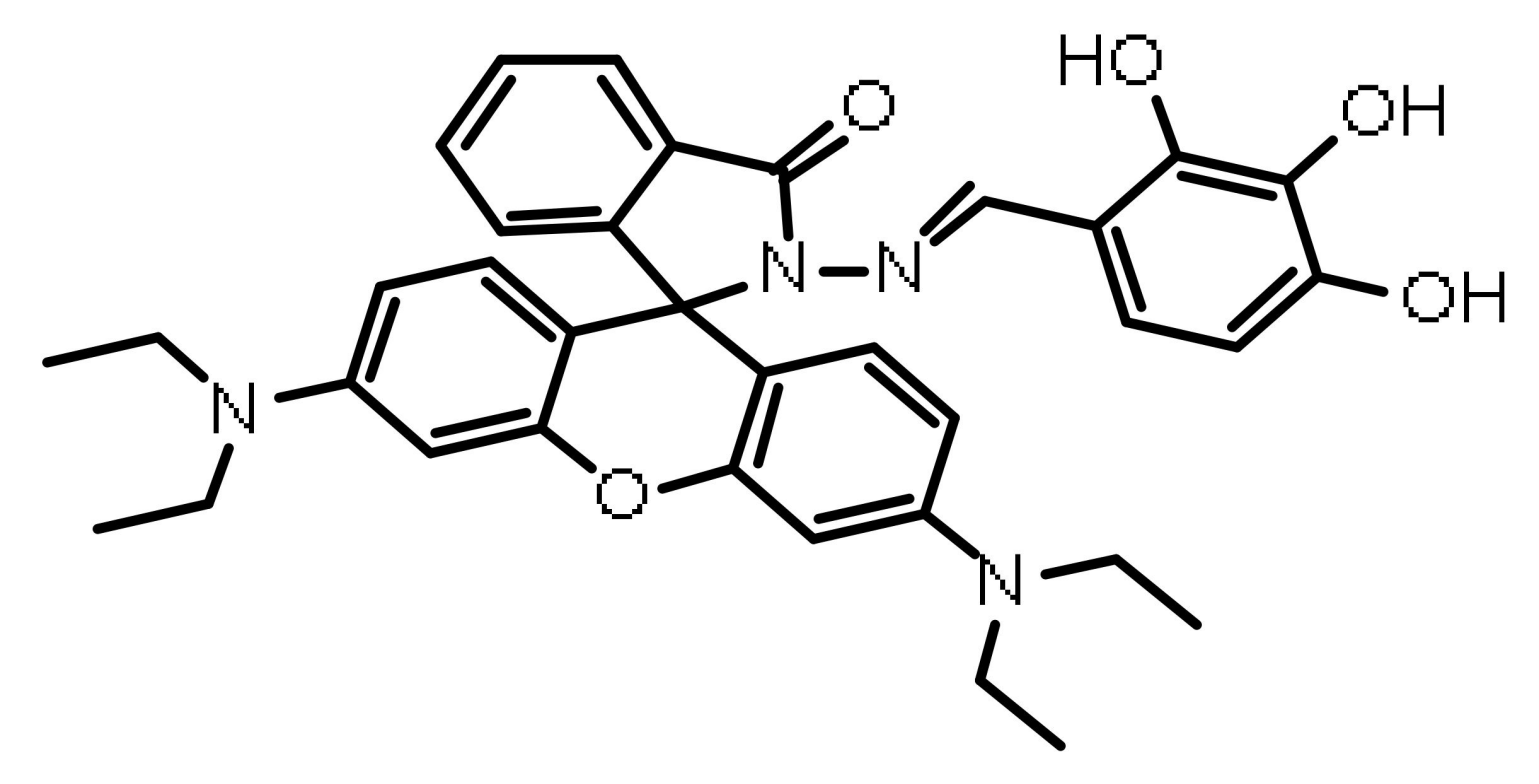	Cu^2+^/Co^2+^	Tris-HCl	0.11/0.88	[53]
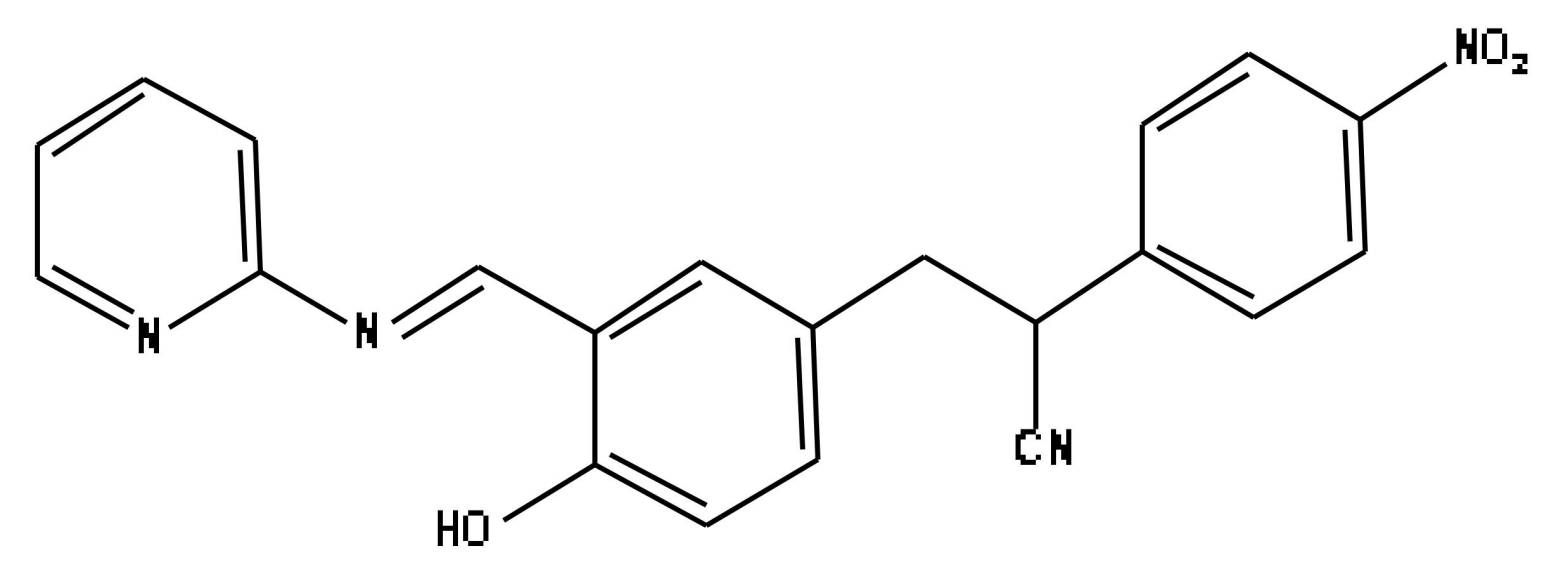	Cu^2+^/Co^2+^	THF/H_2_O (1:99)	0.10/0.09	[54]
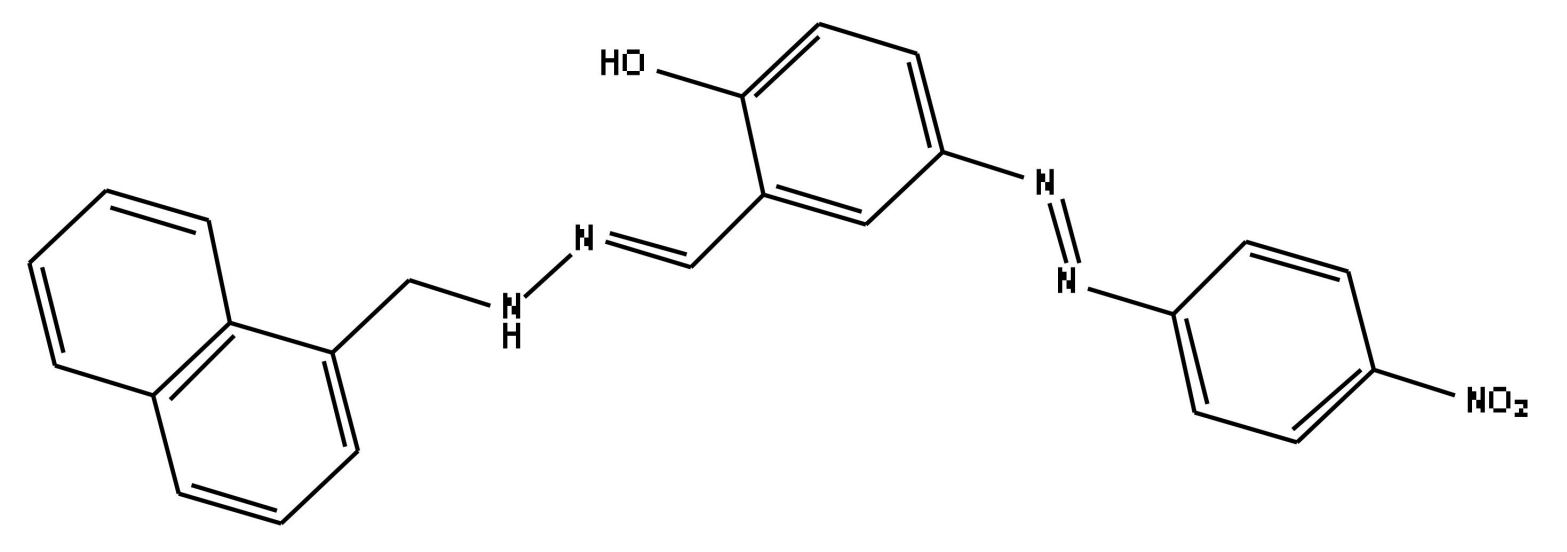	Cu^2+^/Co^2+^	THF/Tris-HCl (9:1)	1.26/2.45	[55]
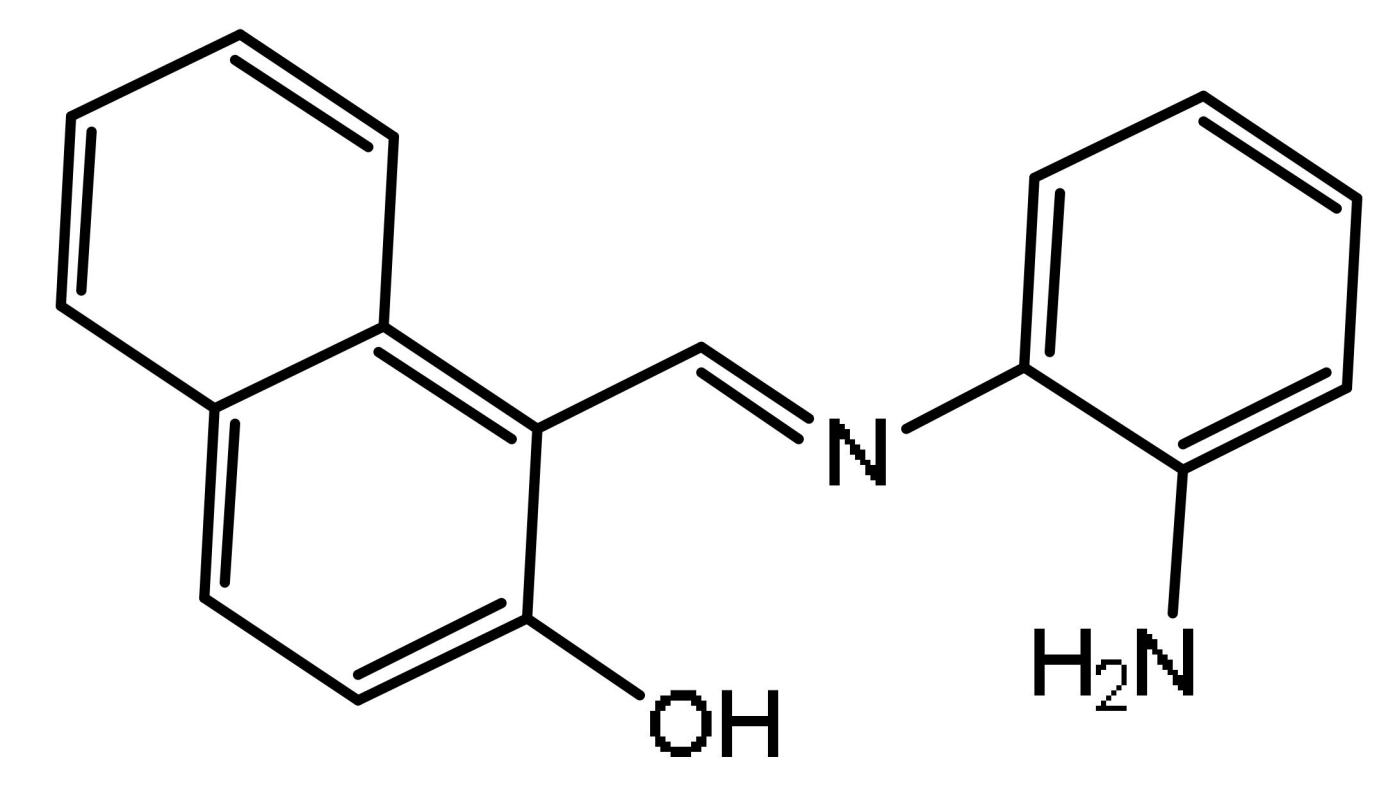	Cu^2+^/Co^2+^	DMSO/H_2_O (3:2)	0.08/0.09	This work

## Data Availability

Not applicable.

## Supplementary Materials

The following supporting information can be downloaded at: https://www.mdpi.com/article/10.3390/molecules28052059/s1, Figure S1: ^1^H-NMR spectrum of probe AMN in (Methyl sulfoxide)-d_6_; Figure S2: ^13^C-NMR spectrum of probe AMN in(Methyl sulfoxide)-d_6_; Figure S3: ESI-MS spectrum of probe AMN; Table S1: Determination of Cu^2+^ in actual samples (n = 3); Table S2: Determination of Co^2+^ in actual samples (n = 3).

## Sample Availability

Samples of the compound AMN are available from the authors.
