# Supplementary material for: A Bifunctional Fluorescence Probe Based on AIE-ICT Strategy for Visual Detection of Cu2+/Co2+ in Complex Matrix
Source: Molecules. 2023 Feb 22;28(5):2059. doi: 10.3390/molecules28052059 (PMC10003869; doi:10.3390/molecules28052059)
Supplement: Supplementary file 1 [file molecules-28-02059-s001.zip › molecules-2181961-supplementary.pdf]

Table S1. Determination of Cu^{2+} in actual samples (n=3)

Sample	Added (μM)	Founded (μM)	Recovery (%)	RSD (%)
Tap water	0	ND	ND	ND
	20	1.99	99.92	1.17
	50	49.19	98.61	1.22
	80	79.28	99.19	0.50
River water	0	ND	ND	ND
	20	1.96	99.58	1.55
	50	51.62	102.77	1.48
	80	79.01	98.88	0.46
Yellow croaker	0	ND	ND	ND
	20	1.99	99.92	1.17
	50	49.23	98.68	1.04
	80	79.30	99.21	0.95

ND: not detected.

Table S2. Determination of Co^{2+} in actual samples (n=3)

Sample	Added (μM)	Founded (μM)	Recovery (%)	RSD (%)
Tap water	0	ND	ND	ND
	20	1.99	99.48	2.55
	50	51.65	103.27	1.09
	80	80.94	101.17	0.44
River water	0	ND	ND	ND
	20	1.99	99.48	2.55
	50	50.81	101.62	0.41
	80	80.49	100.62	0.95
Yellow croaker	0	ND	ND	ND
	20	1.98	99.00	1.33
	50	49.95	99.90	1.37
	80	79.17	98.96	0.79

ND: not detected.

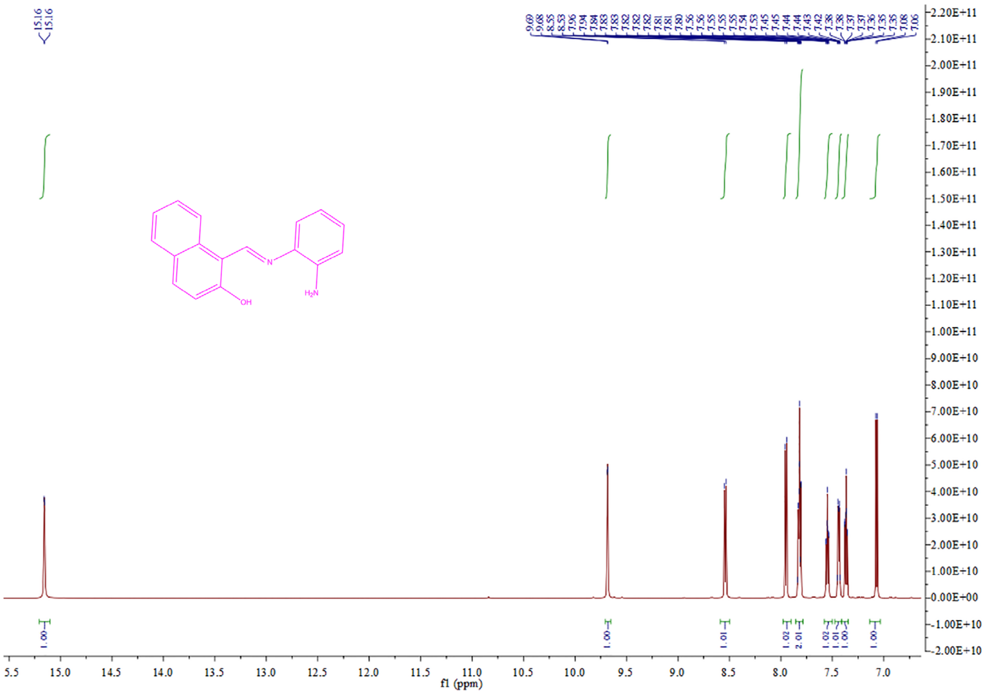

Fig. S1 ^{1}H-NMR spectrum of probe AMN in (Methyl sulfoxide)-d_6.

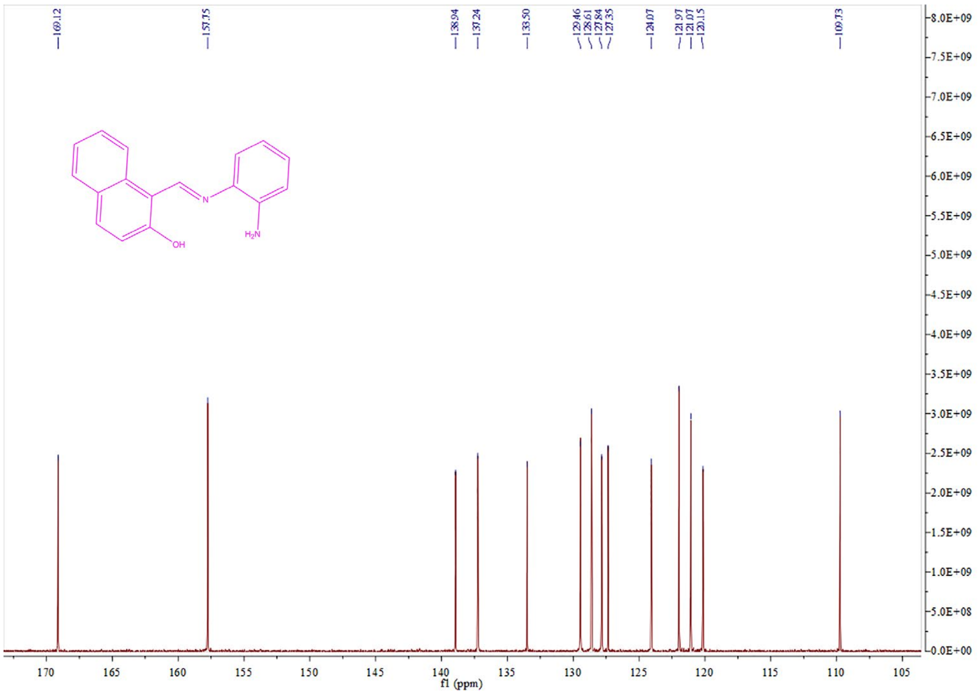

Fig. S2 ^{13}C-NMR spectrum of probe AMN in(Methyl sulfoxide)-d_6.

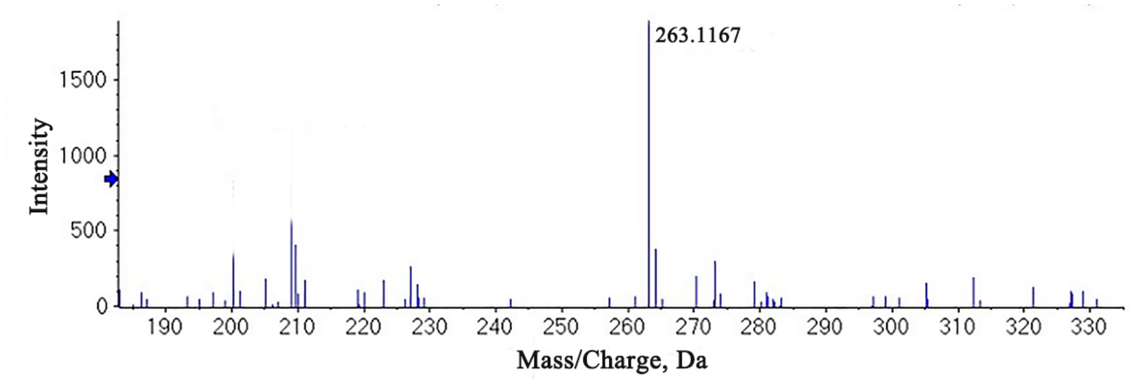

Fig. S3 ESI-MS spectrum of probe AMN.
